# The Survival Rate of Hospitalized Lupus Patients With Overlap Disease

**DOI:** 10.7759/cureus.94336

**Published:** 2025-10-11

**Authors:** Jaafar Al-sadiq Jaf, Seyedeh Tahera Faezi, Amir Kasaeian

**Affiliations:** 1 Stroke, Stepping Hill Hospital, Stockport NHS Foundation Trust, Stockport, GBR; 2 Rheumatology, Rheumatology Research Center, Tehran University of Medical Sciences, Tehran, IRN; 3 Statistics, Research Center for Chronic Inflammatory Diseases, Tehran University of Medical Sciences, Tehran, IRN; 4 Research, Liver and Pancreatobiliary Diseases Research Center, Digestive Diseases Research Institute, Tehran University of Medical Sciences, Tehran, IRN; 5 Research, Digestive Oncology Research Center, Digestive Diseases Research Institute, Tehran University of Medical Sciences, Tehran, IRN

**Keywords:** autoimmune disease, mortality, overlap diseases, survival rate, systemic lupus erythematosus

## Abstract

Objective

Systemic lupus erythematosus (SLE) is a multisystem autoimmune disease associated with a rate of mortality during hospitalization. On the other hand, SLE can be associated with other autoimmune diseases with different morbidity and mortality, so a combination of SLE and other autoimmune diseases, as overlap diseases, may increase the risk of morbidity and mortality. The aim of this study was to determine the survival rate of hospitalized SLE patients who also had other autoimmune diseases.

Method

We used a retrospective data collection from the inpatient files collected over the past 10 years in the Shariati Hospital, Rheumatology Department, Tehran University of Medical Sciences.

Results

We identified 219 patients with SLE who overlapped with other autoimmune diseases. Nearly 90% (n=197) were female patients. The mean age of patients was 37.9 years. The most common overlap autoimmune disease alongside SLE was anti-phospholipid syndrome (APS; n=99; 45.4%), followed by rheumatoid arthritis (RA) and scleroderma (n=29; 13.3% each). We had eight (3.7%) deaths in the hospitalized patients, of which six (75.0%) were female patients. The SLE patients who had overlap with APS, scleroderma, and RA died during hospitalization. The general survival rate of SLE patients with at least one autoimmune disease was around 75% (n=164), and the female patients had a better survival rate than the male patients (n=152; 77.0% and n=12; 75.0%, respectively). Infection was the main cause of mortality.

Conclusion

The general survival rate of SLE patients with at least one autoimmune disease was around 75%. Presence of overlap with APS, scleroderma and RA, and male gender worsened the survival rate.

## Introduction

Systemic Lupus Erythematosus (SLE) is an autoimmune disease in which organs and cells undergo damage, initially mediated by tissue-binding autoantibodies and immune complexes [1]. The prevalence of SLE in Iran is reported as 40 per 100,000 [2]. The main causes of death in SLE are infection, atherosclerotic disease, and organ damage [3]. At least 25% of autoimmune rheumatic diseases present with overlap syndrome with features of more than one disease [4], with defined morbidity and mortality rates. It’s important to analyze the data of patients with overlapping conditions, as such patients may not respond to standard treatment and may develop more comorbidities that may increase morbidity and mortality. So, it’s important to evaluate the outcome (mortality and survival rate) of SLE patients with overlapping autoimmune diseases.

## Materials and methods

In this study, we used retrospective data collected from the inpatient files of SLE patients from 2009 to 2019 at the Shariati Educational Hospital, Tehran University of Medical Sciences (TUMS), Tehran, Iran. The primary aim of our retrospective study was to review the outcomes of hospitalized lupus patients who had an overlap with another autoimmune disease and to assess their survival rates. We reviewed the inpatient files of SLE patients who fulfilled the Systemic Lupus International Collaborating Clinics (SLICC) criteria [5], and collated around 1200 cases. We then conducted a filtering process to identify lupus patients with overlap disease, and obtained 219 cases over the 10 years under consideration. Disease flare was defined based on the treating physician’s documentation and supported by laboratory findings. All patients underwent standard laboratory investigations according to departmental protocols, including antinuclear antibody (ANA), anti-double-stranded DNA (anti-dsDNA), complement levels (C3 and C4), and antiphospholipid antibodies. These tests were consistently performed across all study years, ensuring comparability despite minor technical updates in assay methods.

Inclusion criteria

Patients admitted to the Shariati Educational Hospital between 2009 and 2019 with a confirmed diagnosis of SLE according to the 2012 SLICC classification criteria were eligible. Only those who also met established diagnostic criteria for at least one additional autoimmune rheumatic disease, such as anti-phospholipid syndrome (APS), systemic sclerosis, Sjögren’s syndrome, Behçet’s disease, mixed connective tissue disease, or another systemic autoimmune disorder, were included. Although some patients were initially diagnosed before 2012, all cases were retrospectively re-evaluated according to the 2012 SLICC classification criteria to ensure uniform inclusion across the cohort.

Exclusion criteria

Cases lacking complete medical records, those not fulfilling the SLICC criteria for SLE, or patients without a confirmed overlapping autoimmune diagnosis were excluded.

Statistical analysis

Descriptive statistics, specifically standardized mean difference (SMD) with 95% CI were used to summarize the clinical and serological data. Survival rate percentage of total patients and the survival rate percentage based on gender and overlapped diseases were determined using Kaplan-Meier survival graphs. The cause of death was determined according to the treating physician’s final assessment. Septicemia was confirmed by positive microbiological cultures and clinical evidence of infection, whereas SLE flare was assigned when infection was excluded and lupus activity markers (anti-dsDNA rise, complement reduction) were present. Research ethics committee approval was obtained for the conduct of this study.

## Results

This study included 219 patients, of whom 195 (89.0%) and 24 (11%) were female and male participants, respectively. The majority of our patients were admitted to the rheumatology ward (n=207; 94.5%), and some were admitted to the ICU (n=4; 1.8%). Five (2.3%) patients were admitted to the ICU, then they moved back to the rheumatology ward, after which they were discharged. The mean age of the admitted patients was 38 years, and the mean age at SLE diagnosis was 30 years. The difference between these two values reflects the chronic nature of SLE, where patients are often diagnosed earlier but hospitalized later due to disease flares or overlap-related complications. With respect to causes for admission, almost 68 (30.8%) patients were admitted for SLE disease flare, and the majority had other reasons that were mostly related to the overlapped disease, with 105 (48.0%) patients expressing APS syndromes, mostly vascular thrombosis. Nearly 34 (15.5%) patients had scleroderma signs, and 34 (15.5%) had RA.

A total of eight (3.7%) deaths were recorded among the hospitalized patients, details of which are summarized below. Seven (87.5%) of the eight deaths were associated with septicemia; two patients (25.0%) experienced cardiac arrest, and two (25.0%) had pulmonary embolism as contributing causes of death. Six were female patients (6/8, 75%), while two were male patients (2/8, 25.0%), with an average age of nearly 40 years. Half of the patients who died were admitted to the ICU ward (4/8, 50.0%). Five of the female patients had septicemia (5/6, 83.3%), and one had a cardiac shock as the main cause (1/6, 16.7%) of death. One male patient had septicemia as the main cause of death (1/2, 50.0%) while the other had a SLE disease flare that led to cardiac arrest as the main cause (1/2, 50.0%).

Based on the files of the patients reviewed, the most common systemic rheumatic diseases that overlapped with SLE are shown in Table 1, with APS showing maximum overlap, followed by scleroderma and RA.

**Table 1:** Systemic rheumatic diseases that overlapped with systemic lupus erythematosus (SLE) APS: Anti-phospholipid syndrome; RA: Rheumatoid arthritis; PM/DM: Polymyositis/Dermatomyositis; MCTD: Mixed connective tissue disease.

Overlap	N (%)
APS	99 (45.4%)
Scleroderma	29 (13.3%)
RA	29 (13.3%)
PM/DM	24 (11.0%)
More than 2 rheumatological diseases	16 (7.3%)
Sjogren	12 (5.5%)
MCTD	4 (1.8%)
Ankylosing spondylitis	2 (0.9%)
Other	2 (0.9%)
Behcet	1 (0.5%)

According to the date of admission and the date of discharge we calculated the mean follow-up time of our patients in hospital (12 days). Table 2 shows the mean follow-up time according to the overlap disease.

**Table 2:** Follow-up time in hospital, as per the overlapping disease APS: Anti-phospholipid syndrome; RA: Rheumatoid arthritis; PM/DM: Polymyositis/Dermatomyositis; MCTD: Mixed connective tissue disease.

Comorbidity	Mean follow-up time (days)
APS	14
Scleroderma	14
Sjogren	14
Patients with more than 2 rheumatological diseases	14
RA	10
PM/DM	10
Ankylosing spondylitis	7
MCTD	6
Other	6

The patient data showed that 31 (14.1%) patients had disease onset and symptoms 10 years before their latest admission, 25 (11.5%) at four years prior, whereas 22 (10.1%) patients each had onset of the disease five years and one year earlier.

Kaplan-Meier survival estimates of the study showed that patients who had SLE overlapping with APS, scleroderma, and RA had death and survival rates below 100%. According to the data collected, survival rates of lupus patients with at least one rheumatological disease were a little over 75% (164/219). After further filtering of the patient’s data, we observed that the survival rates in male patients were 75% (12/16), and were slightly lower than that of female patients (76.5%; 152/199), but the difference was not significant. As detailed earlier, the three overlap diseases with a record of death were APS, scleroderma, and RA. Hence, we calculated the survival rate based on the admission time of the patient. We determined that for patients with APS, the survival rates were 100% (99/99) at week one, but as time passed, they went down to 89% (88/99).

**Figure 1:**
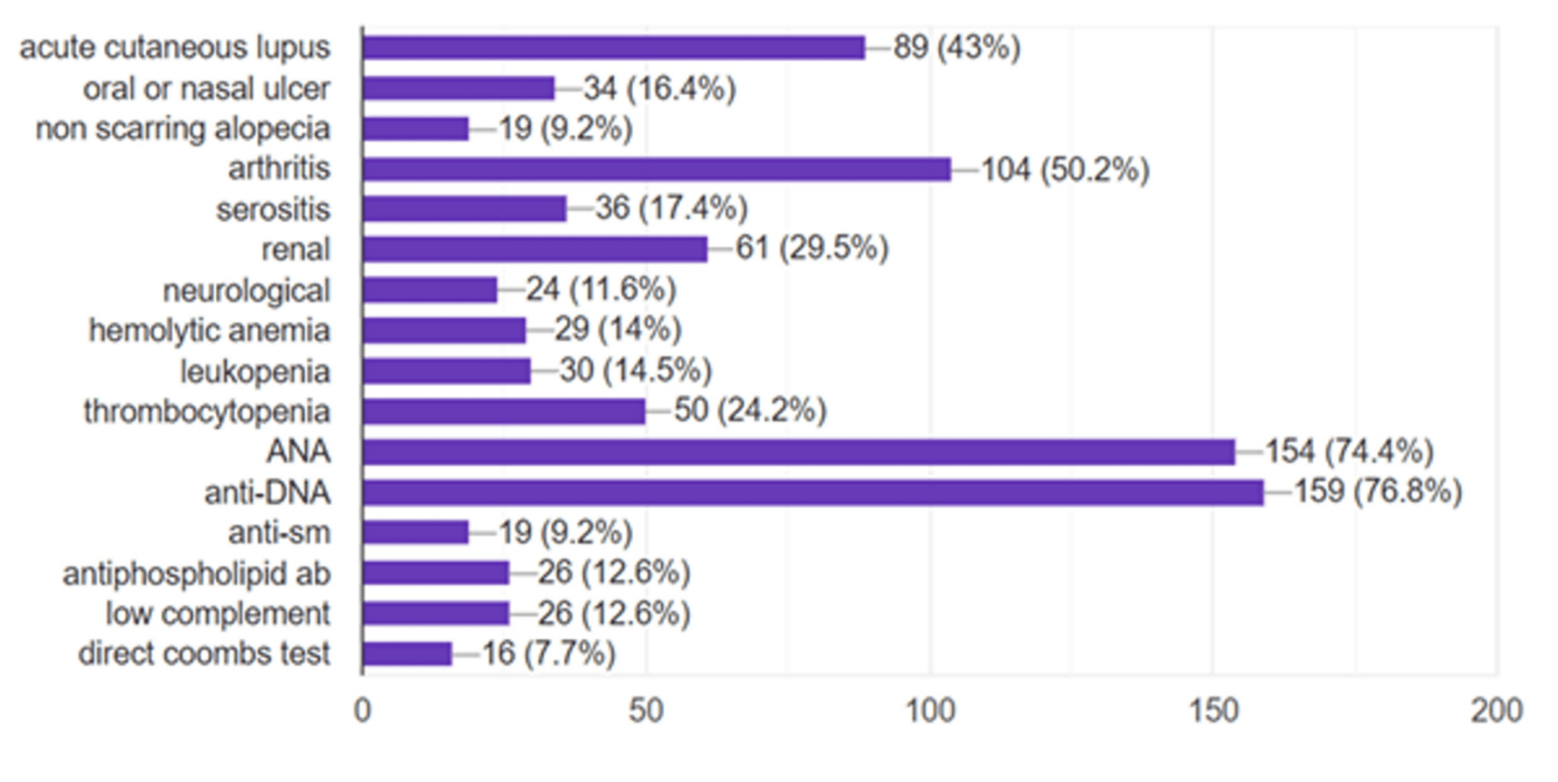
Percentage of SLICC criteria in the group of patients studied (n=219) ANA: Antinuclear antibody; anti-DNA: Anti–double-stranded DNA antibody; anti-SM: Anti-Smith antibody; ab: Antibody; C3/C4: Complement components 3 and 4; SLICC: Systemic Lupus International Collaborating Clinics

Evaluation of the SLICC criteria among our patients showed that the most frequent features were positive fluorescent antinuclear antibody (FANA) and anti-dsDNA tests (both present in >70% of patients), followed by arthritis and cutaneous lupus manifestations. The rest of the criteria are outlined in the graph below (Figure 1).

Further details of the study regarding the drugs used in the month prior to admission, drugs used six months prior to admission, and laboratory findings are presented in Tables 3, 4, and 5, respectively. 

**Table 3:** Drugs used in the month prior to admission

Drug	N (%)
Prednisolone	175 (80.0%)
Hydroxychloroquine	103 (47.0%)
Methotrexate	37 (17.0%)
Azathioprine	46 (21.0%)
Mycophenolate mofetil	15 (6.9%)
Cyclosporin	6 (2.7%)
Methylprednisolone	22 (9.9%)

**Table 4:** Drugs used in the six months prior to admission

Drug	N (%)
Methylprednisolone	21 (9.4%)
Cyclophosphamide	24 (10.9%)
Rituximab	2 (1.0%)

**Table 5:** Findings of laboratory tests CRP: C-reactive protein; ESR: Erythrocyte sedimentation rate; LA: Lupus anticoagulant; FANA: Fluorescent antinuclear antibody; RO: Anti-Ro/SSA antibody; dsDNA: Double-stranded DNA; C3/C4: Complement components 3 and 4.

Test	N (%)
Abnormal liver tests	68 (31.0%)
High glucose	54 (24.7%)
High lipid profile	34 (15.4%)
Anti-dsDNA	155 (71.0%)
Anti-cardiolipin	118 (54.0%)
Lupus anticoagulant	83 (37.8%)
Anti-RO	109 (49.8%)
Proteinuria	72 (32.8%)
>3500 mg	37 (16.9%)
1000–3500 mg	104 (47.7%)
<1000 mg	~77 (>35%)
Low C3	84 (38.2%)
Low C4	85 (38.7%)
Leukopenia	50 (22.6%)
Chronic disease anemia	122 (55.5%)
Hemolytic anemia	32 (14.7%)
Thrombocytopenia	56 (25.5%)
High creatinine	43 (19.5%)
CRP positive	101 (46.0%)
ESR elevated	173 (78.8%)

## Discussion

In this study, we performed a retrospective cohort investigation, including 219 patients admitted to the Shariati Hospital, TUMS, at any time between 2009 and 2019. Our results showed that the survival rate of SLE patients with one overlap disease was 171/219 (78.0%), with male patients having worse survival rates than female patients. In this study, APS was the most common overlap disease with SLE, and mortality occurred only in patients who had overlap with APS, scleroderma, and RA. In our study, 89% (195/219) were female patients, unlike the study conducted in Mexico City, in which all those with SLE overlapping with another disease were female patients [6]. This difference might be explained by the female/male ratio of the population in Mexico, which is higher than in Iran by almost 5%, as well as by genetic differences [7].

The mean age of patients in our study was 37.9 years, whereas in the Mexican study, they were all above 42 years [6]. Regarding death rates, our study showed that the mortality rate over the last 10 years was 3.6%, which is lower than similar studies that reported a mortality rate of 7.9% [6]. One difference between our study and theirs was that their research included patients from 1979 to 2011, covering more than 32 years, which may explain the higher mortality rate [6]. In our study, the main cause of death was infection, followed by cardiac events, and lastly, SLE disease flare. In contrast, the previous study found disease manifestation as the leading cause of death, followed by septicemia and cardiac events [6]. Compared to known SLE mortality causes, which include infection, atherosclerotic disease, and organ damage [2], we observed that infection and cardiac manifestations were common to both.

Our results showed that most of our lupus patients had overlap with APS (99/219; 45.4%), followed by scleroderma (29/219; 13.3%) and RA (29/219; 13.3%), and then Sjogren’s syndrome (12/219; 5.5%). Mortality was recorded only in patients with the first three overlap diseases. When we compared our results to a study from New York, USA, we found that APS prevalence was similar (43%), but our rates of Sjogren’s and RA were lower, whereas that study reported 52% and 30% for both conditions, respectively. They also had a higher mean age and an almost equal proportion of male patients compared to our study. This difference can be explained in several ways, but a key point is that RA and Sjogren’s are more common in Eastern European and European countries. Additionally, the large difference in the percentage of Sjogren’s may be because, unlike most autoimmune disorders, it lacks universally accepted classification criteria [8].

**Figure 2:**
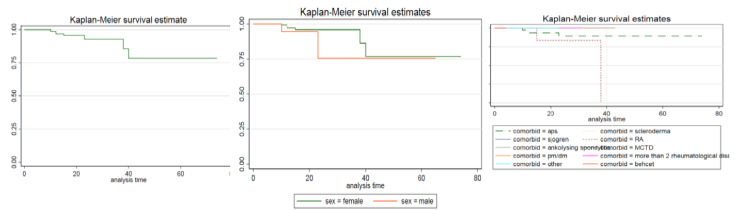
Kaplan-Meier survival estimates

Based on the Kaplan-Meier analysis, the overall in-hospital survival rate was approximately 75-78%, consistent across both descriptive and survival analyses (Figure 2).

When compared to a USA-based study of SLE patients (not specifically overlap cases), the Kaplan-Meier estimates were greater, with a survival rate of 78% (female patients at 79% and male patients at 68%). In both studies, male survival was lower than female survival. The slight differences between our results and theirs can be attributed to the fact that their research reviewed all SLE patients, not just those with overlap diseases [9]. For more precise conclusions, a large-scale study including all SLE patients would be beneficial.

Based on the patient data we collected, 20.1% (44/219) of our patients had not used prednisolone, and this percentage increased to 37.5% (3/8) among the deceased patients. Around 10% (22/219) had received methylprednisolone. This can be compared to a similar study in which 11% of patients had not used prednisolone and 19% had received methylprednisolone [10]. However, their research included all SLE patients, unlike ours which focused only on SLE with overlap.

Our study also showed that 17.9% (39/219) of patients used methotrexate, 21.5% (47/219) used azathioprine, and 14.2% (31/219) used cyclophosphamide. As reported by Pistiner et al., methotrexate usage was around 5%, azathioprine was 13%, and cyclophosphamide was 14% [11].

In the same study on lupus patients, leukopenia was found in 54%, positive anti-DNA in 40%, chronic disease anemia in 30%, hemolytic anemia in 8%, high lipid profile in 21%, low C3 in 39%, low C4 in 29%, positive RO in 18%, and positive lupus anticoagulant (LA) in 2% [12]. In our study, leukopenia occurred in 22.6% (50/219), positive anti-DNA in 70.8% (155/219), anemia of chronic disease in 55.7% (122/219), hemolytic anemia in 14.7% (32/219), high lipid profile in 15.5% (34/219), low C3 in 38.4% (84/219), and low C4 in 38.8% (85/219). It is important to note that the other study was conducted in 1980, with different diagnostic criteria and laboratory methods, which may explain some of the variations [11].

In a Danish study on SLE patients, 29% had proteinuria, whereas in our study, 32.8% of patients experienced proteinuria. The Danish study focused on SLE patients without overlap, whereas in our research, the overlap may have contributed to a higher risk of organ damage [13].

Limitations

This study has several limitations. It was conducted in a single tertiary care center and included only hospitalized patients, which may not fully represent the broader population of SLE patients with overlap diseases. The retrospective design may have led to missing or incomplete data, particularly in older patient files. In addition, some overlap subgroups had a small number of cases, which limited the statistical power of the subgroup analysis. Finally, survival outcomes were assessed only during hospitalization and not in long-term follow-up, which restricts conclusions about the overall prognosis. Although this study covered a 10-year period, the rheumatology department at the Shariati Hospital maintained consistent care standards throughout. However, gradual improvements in diagnostic tools and management protocols over time may have introduced minor variability in patient outcomes. Advances in treatment strategies and supportive care over the study period may also have contributed to improved survival outcomes. However, core therapeutic protocols remained largely consistent.

## Conclusions

This study emphasizes that SLE patients with overlapping autoimmune or rheumatic diseases represent a particularly vulnerable subgroup with more complex disease presentations and outcomes during hospitalization. The coexistence of additional autoimmune disorders appears to increase clinical complications, making disease management and treatment decisions more challenging. These patients often present with multiple overlapping immunological mechanisms that may contribute to more severe organ involvement and variable therapeutic responses.

In this context, overlaps involving APS, RA, and scleroderma were especially associated with poorer in-hospital outcomes. Male patients also tended to experience less favorable survival patterns compared to female patients. These findings highlight the need for early recognition of overlap features in lupus patients, personalized treatment strategies, and close monitoring for infection and organ involvement-factors that remain critical to improving both short-term and long-term outcomes in this high-risk group.
