# Correction: The Survival Rate of Hospitalized Lupus Patients With Overlap Disease

**DOI:** 10.7759/cureus.c364

**Published:** 2025-10-16

**Authors:** Jaafar Al-sadiq Jaf, Seyedeh Tahera Faezi, Amir Kasaeian

**Affiliations:** 1 Stroke, Stepping Hill Hospital, Stockport NHS Foundation Trust, Stockport, GBR; 2 Rheumatology, Rheumatology Research Center, Tehran University of Medical Sciences, Tehran, IRN; 3 Statistics, Research Center for Chronic Inflammatory Diseases, Tehran University of Medical Sciences, Tehran, IRN; 4 Research, Liver and Pancreatobiliary Diseases Research Center, Digestive Diseases Research Institute, Tehran University of Medical Sciences, Tehran, IRN; 5 Research, Digestive Oncology Research Center, Digestive Diseases Research Institute, Tehran University of Medical Sciences, Tehran, IRN

This article has been revised to change the affiliation for Amir Kasaeian. The previous affiliation was: ​​ 

Statistics, Hematology, Oncology and Stem Cell Transplantation Research Center, Tehran University of Medical Sciences, Tehran, IRN 

This has been updated to:

1. Research, Research Center for Chronic Inflammatory Diseases, Tehran University of Medical Sciences, Tehran, Iran.
2. Research, Liver and Pancreatobiliary Diseases Research Center, Digestive Diseases Research Institute, Tehran University of Medical Sciences, Tehran, Iran.
3. Research, Digestive Oncology Research Center, Digestive Diseases Research Institute, Tehran University of Medical Sciences, Tehran, Iran.

